# I-124 Imaging and Dosimetry

**DOI:** 10.4274/2017.26.suppl.07

**Published:** 2017-01-09

**Authors:** Russ Kuker, Manuel Sztejnberg, Seza Gulec

**Affiliations:** 1 University of Miami Miller School of Medicine, Department of Radiology, Miami, USA; 2 National Atomic Energy Commission of Argentina, Division of Instrumentation and Dosimetry, Buenos Aires, Argentina; 3 Florida International University Herbert Wertheim College of Medicine, Departments of Surgery and Nuclear Medicine, Miami, USA

**Keywords:** I-124 positron emission tomography/computed tomography, dosimetry, thyroid cancer

## Abstract

Although radioactive iodine imaging and therapy are one of the earliest applications of theranostics, there still remain a number of unresolved clinical questions as to the optimization of diagnostic techniques and dosimetry protocols. I-124 as a positron emission tomography (PET) radiotracer has the potential to improve the current clinical practice in the diagnosis and treatment of differentiated thyroid cancer. The higher sensitivity and spatial resolution of PET/computed tomography (CT) compared to standard gamma scintigraphy can aid in the detection of recurrent or metastatic disease and provide more accurate measurements of metabolic tumor volumes. However the complex decay schema of I-124 poses challenges to quantitative PET imaging. More prospective studies are needed to define optimal dosimetry protocols and to improve patient-specific treatment planning strategies, taking into account not only the absorbed dose to tumors but also methods to avoid toxicity to normal organs. A historical perspective of I-124 imaging and dosimetry as well as future concepts are discussed.

## Abstract (tr)

Radyoaktif iyot görüntüleme ve tedavisi teranostiğin ilk uygulamalarından biri olmasına rağmen, halen tanı teknikleri ve dozimetri protokollerinin optimizasyonu ile ilgili çözümlenmemiş klinik sorular bulunmaktadır. Pozitron emisyon tomografi (PET) radyofarmasötiği olarak I-124, mevcut klinik uygulamada diferansiye tiroid kanserlerinin tanı ve tedavisini geliştirme potansiyeline sahiptir. Standart gamma sintigrafisi ile karşılaştırıldığında PET/bilgisayarlı tomografinin yüksek duyarlılık ve uzaysal çözünürlüğü nüks veya metastatik hastalığın saptanmasında yardımcı olabilir ve metabolik tümör hacimlerinin daha doğru ölçülmesini sağlayabilir. I-124’ün karmaşık bozunma süreci kantitatif PET görüntüleme için zorluklar teşkil etmektedir. Optimum dozimetre protokollerini tanımlamak ve hastaya özgü tedavi planlama stratejilerini sadece tümör tarafından abzorbe edilen doza değil normal organlara toksisiteyi önleyecek metotlara dayanarak iyileştirecek prospektif çalışmalara ihtiyaç vardır. I-124 görüntüleme ve doz ölçümünün tarihsel perspektifi ile gelecekteki kavramlar tartışılmıştır.

## INTRODUCTION

The mainstay of current clinical radioactive iodine (RAI) imaging and dosimetry utilizes the radioisotope I-131. The physical half life of I-131 is 8.02 days that allows imaging and data acquisition at sequential time points over a period of several days. The high energy gamma emissions of 364 keV, however, are a drawback due to poor image quality resulting in less than optimal evaluation of extent of disease and quantitation. Another factor contributing to poor image quality is the relatively small administered activities in the range of 2-5 mCi. The clinical safety/efficacy of higher administered activities of I-131 has been questioned due to concern for the “stunning effect” when it is used prior to RAI treatment. I-131 remains the standard treatment for differentiated thyroid cancer (DTC) because of its beta emissions with a maximum energy of 606 keV and tissue penetration of 0.6-2 mm.

I-123 is predominantly a gamma emitter with an energy of 159 keV, which is near the optimal range for standard gamma camera imaging. In addition, higher administered activities can be used as compared to I-131 because of less concern for stunning. Although the physical properties of I-123 contribute to improved image quality, there are also several drawbacks. I-123 is is more expensive and is not as widely available as I-131. The absence of beta decay does not permit the use of I-123 for therapeutic applications, and the relatively short half life of 13 hours limits its utility for dosimetry protocols.

I-124 is a PET radiopharmaceutical with a 4.2-day half life. It offers superior imaging characteristics with enhanced spatial resolution and image sensitivity due to coincidence detection on PET cameras. I-124 also has a favorable half life that permits the evaluation of in-vivo iodine kinetics. However, I-124 has a rather complex decay schema, which creates challenges in optimal imaging. There exist a number of reports attesting the potential clinical benefits of I-124 imaging in patients with DTC, however, a uniform clinical protocol for imaging, image analysis and quantitation has not yet been firmly established.

## HISTORY OF I-124 IN CLINICAL PRACTICE

The use of I-124 in clinical practice in a patient with DTC was first described in 1960 (1), and the first PET-based dosimetry study based on serial measurements in DTC patients was published in 1999 (2) after phantom studies demonstrated that quantification was feasible (3). Since then, there have been several key articles demonstrating the clinical utility of I-124 and its role in the detection of residual thyroid tissue and/or metastasis in patients with DTC (4,5,6,7,8); and the following are some more recent highlights.

In 2010, Van Nostrand et al. (6) compared the ability of I-124 PET versus I-131 planar whole body imaging in detecting residual thyroid tissue and/or metastatic DTC. Twenty-five patients were included in the study. Eight of the 25 patients showed more positive foci of uptake on I-124 images than on I-131. I-124 demonstrated the same number of foci as on I-131 in 16 patients. One patient had one additional positive focus on I-131 not seen on I-124, which could not be confirmed as a metastasis. Out of 97 positive foci, I-124 identified 49 that were not seen with I-131, and I-131 identified one positive focus not seen with I-124. They concluded that relative to I-131 planar whole body imaging, I-124 PET identified as many as 50% more foci of radioiodine uptake suggestive of residual thyroid tissue and/or metastases in as many as 32% more patients who had DTC.

In 2015, as part of the THYROPET study, Kist et al. (7) focused on a subset of patients with suspected recurrence from DTC, based on an increased thyroglobulin level and negative neck ultrasound. All patients underwent I-124 PET/CT after rhTSH stimulation. Subsequently, after 4-6 weeks of thyroid hormone withdrawal, the patients were treated with 150-200 mCi of I-131 and underwent a planar whole body scan (WBS) one week later. The study was terminated preliminarily after inclusion of 17 patients. Eight post-therapy WBS were negative (47%), all of which were correctly predicted by a negative I-124 PET/CT. Nine post-therapy WBS showed RAI avid tumor, of which only four also had positive I-124 PET/CT findings. The authors observed that 29% of patients (5/17) would have been denied potentially effective RAI therapy if I-124 PET/CT was implemented. Of the false negative lesions with anatomical correlates on CT, the majority were located in the lungs. They concluded that the high false negative rate of rhTSH stimulated I-124 PET/CT precludes its use as a scouting procedure in this cohort of patients. The authors postulate that their observed false negative rate may have been influenced by the 2 mCi dose of I-124 as compared to the higher doses of I-131 and the use of rhTSH instead of thyroid hormone withdrawal.

In 2016, our group conducted a phase I/II study to determine the imaging characteristics and clinical feasibility of I-124 PET/CT for the determination of disease extent and evaluation of RAI kinetics in its physiologic and neoplastic distribution in patients with DTC (8). Fifteen patients were included in the study. All patients who had I-124 imaging subsequently underwent RAI treatment with administered activities of I-131 in the range of 100 to 300 mCi. Post-treatment planar whole body and static images were obtained 5 to 7 days after RAI treatment and used as a reference to compare with the pre-treatment I-124 PET/CT scans. Forty-six distinct lesions were identified in these 15 patients on I-124 PET/CT images with a sensitivity of 92.5%. I-124 identified 22.5% more foci of RAI avid lesions as compared to the planar I-131 post-treatment scans. In addition, the I-124 images provided discriminating details in terms of location and laterality of remnant thyroid tissue even when no remnant tissue was appreciated on anatomical imaging. Our group concluded that I-124 PET/CT is a valuable clinical imaging tool/agent, in both extent of disease evaluation in the setting of metastatic DTC and the functional volumetric and kinetic evaluation of target lesions.

## I-124 PROPERTIES AND DECAY SCHEMA

I-124 is cyclotron produced and because of its 4.2-day half life can be easily transported for clinical use. I-124 has a positron abundance (number of positrons emitted per decay) of only 23% with maximum and mean positron energies of 2138 and 819 keV, respectively. In contrast, the most common PET radiotracer, F-18, has a positron abundance of 97% with maximum and mean positron energies of 634 and 250 keV, respectively. It is estimated that this high positron emission energy results in a spatial resolution loss of about 1 mm compared to F-18 FDG which has a spatial resolution on the order of 5-10 mm depending on the scanner and clinical reconstruction parameters (3).

In addition to positron emissions, I-124 emits a rather large portion of gamma rays during its decay, over half of which have an energy of 603 keV (Table 1). Coincidences of this 604 keV photon and a 511 keV annihilation photon cannot be distinguished from the true coincidences involving two 511 keV annihilation photons. These gamma coincidences result in background activity on the PET images which has also been described for other PET radiotracers with longer half lives such as Y-86 and Br-76 (9,10). Multiple correction methods have been suggested to address this background activity but their effectiveness is limited in the setting of the low count rates observed on clinical I-124 scans. In addition, the large amount of gamma emissions leads to higher random coincidence rates, increased dead time and inaccurate dead time correction (10). Improved gamma coincidence correction and dead time correction methods are necessary to ensure accurate quantitative imaging of I-124 (11).

## BACKGROUND OF RADIOACTIVE IODINE DOSIMETRY

The general principle of RAI dosimetry relies on the premise that administered activities for diagnostic or therapeutic purposes should be as low as reasonably achievable to limit the radiation burden to the individual as well as the general population. RAI treatment is one of the earliest applications of theranostics and is certainly a strong therapeutic intervention, along with surgical treatment, in the management of DTC. The term “ablation” is used when RAI is given to destroy residual thyroid tissue in the absence of functioning thyroid cancer. The term “therapy” refers to administration of RAI for residual, recurrent or metastatic DTC. Although RAI therapy is generally well tolerated, serious side effects can occur and may be deterministic (severity increases with dose and threshold dependent) or stochastic (occur by chance and non-threshold dependent). Deterministic effects of RAI therapy can be early and usually transient (i.e. gastritis, sialadenitis, hypospermia or amenorrhea) or late (i.e. sicca syndrome, lung fibrosis or bone marrow depression). Secondary malignancy is the main stochastic effect of RAI therapy although the incidence is relatively low and comparable to other modalities such as external radiotherapy.

The activity to be used for RAI therapy remains a subject of discussion. Although an empiric fixed-dose RAI treatment protocol is still in common use, the therapeutic profile of RAI is best achieved by application of dosimetry-guided, patient-specific treatment planning. Two approaches for RAI dosimetry have been applied in clinical practice: the maximum safe dose approach which was first introduced by Benua and Leeper and is aimed at administering the highest amount of I-131 which does not result in bone marrow suppression (<2 Gy absorbed dose to the bone marrow with a whole body retention <4.44 GBq at 48 hours) (12,13) and the lesion-based dosimetry method based on the data by Maxon et al. (14,15) in which a target dose (at least 80 Gy to metastatic sites and 300 Gy to thyroid remnants) is calculated on the basis of planar diagnostic I-131 scanning.

The maximum safe dose approach focuses on the safety of RAI treatment. Blood is used as a surrogate of the red bone marrow, which is considered as the critical organ in this approach. Over the years, the original Benua method was refined with improved patient specificity using Medical Internal Radiation Dose (MIRD) methodology. Strengths of the blood-based dosimetry approach include determination of the maximum safe activity of RAI for each individual patient, identification of patients for whom empiric fixed activities are not considered safe, and the potential to administer higher activities at once instead of multiple fractions of lower activity in order to avoid changes in tumor/lesion biokinetics. Although there is a long history of using this treatment strategy at multiple institutions, there is a paucity of clinical data to show improved response or outcome rates. In addition, the absorbed dose to the tumor is not known in this approach and although controversial, there is a risk of stunning with dosimetric administrations of I-131 that may alter tumor/lesion biokinetics during subsequent treatment (16).

The objective of lesion-based dosimetry is to determine the RAI activity that delivers the recommended absorbed dose of radiation to ablate thyroid remnants or treat metastatic disease while minimizing the risk to patients. The calculation of lesion dose is generally based on MIRD methodology and for smaller lesions the spherical model of OLINDA/EXM can be employed. In lesion-based dosimetry, not only is it important to determine how much activity is contained within the lesion, it is also necessary to calculate the mass of the lesion. This can be challenging especially for ablation purposes as measuring the size of thyroid remnants is often not reliable using anatomical imaging such as ultrasound or computed tomography (CT). Some disadvantages of a lesion-based approach to RAI treatment include the wide range of absorbed doses to lesions within a given patient and the inhomogeneous absorbed dose distributions and lack of accurate models to reflect RAI kinetics within individual lesions (16). Since the accuracy of all dosimetric approaches relies on the accurate quantitation of RAI kinetics, I-124 being a PET radiotracer with enhanced spatial resolution may play a pivotal role in this area.

## I-124 DOSIMETRY PROTOCOLS AND CLINICAL APPLICATIONS

Why is I-124 well-suited for RAI dosimetry? Of the available radio-iodine isotopes, I-123 would be the preferred agent for dosimetry given its photopeak that is close to the optimal range for gamma camera imaging; however, its relatively short half life precludes imaging at sequential time points in a practical manner. I-131 is relatively inexpensive, is widely available and has become the mainstay of RAI dosimetry. However, only small administered activities can be given because of the risk of stunning. Additionally the high energy gamma emissions result in poor image quality. I-124 behaves biochemically similar to I-131 and its physical half life of 4.2-days makes it suitable for sequential time point imaging and absorbed dose calculations. With I-131 planar imaging, there is uncertainty regarding RAI avid lesion dimensions, and with single-photon emission computed tomography (SPECT), the counting rate at diagnostic activity levels is inadequate for accurate quantification. Given the added benefit of PET/CT with I-124, not only can fine details be discriminated that are not visible on low-dose planar imaging but also areas of radiotracer uptake can be detected that do not have a measurable anatomical correlation on CT (such as with thyroid remnants as observed in our study). However, there are drawbacks of using I-124 for RAI dosimetry including its relatively high cost and complex decay schema, which may lead to background noise and voxel oversaturation even at low administered activities.

Several investigational studies have successfully used I-124 PET alone and with CT to guide postsurgical treatment and, in particular, RAI therapy in patients with DTC. The number of measurement time points varies for each I-124 dosimetry protocol described in the literature (17,18,19,20,21,22,23). An important observation that should be highlighted is the high intra- and inter-lesional variability in RAI avid tumors (18) as well as in normal tissues such as salivary glands (24,25).

Our group demonstrated different kinetic profiles for normal thyroid remnants, salivary glands, and metastatic lesions as well as individual variations in functional volumes, and thus cumulated activities (8). The sequential I-124 PET/CT images consistently demonstrated the maximum activity within thyroid remnant tissue to occur at 24 hours. After the peak activity was reached, the clearance was mono-exponential. The maximum remnant activity ranged from 0.044 to 7.988 MBq with the total functional remnant volume (the total number of voxels within the remnant ROI) ranging from 1 to 60 ml. Physiologic activity within the salivary glands reached a peak at 4 hours after radioiodine administration. The salivary gland clearance was bi-exponential with an average of 81% of the activity being cleared from the salivary glands by 24 hours. In contrast, metastatic disease to lymph nodes demonstrated an uptake pattern that was significantly different than the thyroid remnant or physiologic salivary gland activity. A progressive increase in activity with protracted retention was identified as a characteristic pattern for metastatic nodal disease. The remnant data is particularly interesting as it again highlights the potential value of individualized patient-specific dosimetric assessment even for purposes of thyroid remnant ablation rather than an empiric or “one dose fits all” approach. However, a larger scale remnant dosimetry study is required to address this issue.

Why do we need RAI dosimetry? Although many centers have adapted a fixed-dose of I-131 in the range of 100-200 mCi for the treatment of metastatic DTC, there are several drawbacks to using this empiric technique. Without knowledge of the rate of radiobiological clearance as well as the degree of intra-lesion variability in absorbed dose, a proportion of patients will be over- or under-treated using an empiric dose of I-131. Benua et al. (12) observed that repeated sub-therapeutic doses of RAI might induce dedifferentiation and loss of iodine-concentrating ability of tumors. The rationale of using the highest possible dose is based on the radiobiologic fact that radiation treatment efficacy is directly related to the radiation dose delivered. Therefore, the goal of dosimetry-guided RAI therapy is to derive a patient-specific dose that will deliver the highest possible tumoricidal effect to metastatic sites while minimizing toxicity to normal tissues. Another potential application of RAI dosimetry involves the ablation of thyroid remnants where the goal is to deliver the lowest possible ablative dose taking into account patient-specific factors such as the functional volume of thyroid tissue remaining as well as the variability in dose distribution.

## TECHNICAL CONSIDERATIONS OF I-124 DOSIMETRY

Traditional I-131 dosimetry using MIRD methodology involves sequential time point whole body imaging and blood sampling to obtain time activity curves and residence times for the whole body and bone marrow compartments. The classic formula D=xS, takes into account the cumulated activities derived from both compartments incorporating gamma emissions from the whole body to the bone marrow and beta emissions from the bone marrow to the bone marrow. The S values are obtained from anthropomorphic phantoms and assume uniform distribution of activity within a source organ of defined geometry.

Our group applied the MIRD schema to define a protocol for I-124 dosimetry. The protocol involves acquiring whole body PET/CT images and blood samples at 4 different time points, i.e. 4, 24, 48 and 72 hours following the administration of a 2 mCi dose of I-124. Regions of interest were drawn around the whole body at each time point in order to generate a time activity curve for the whole body compartment. Similarly, I-124 counts were measured from the blood samples at each sequential time point to generate a time activity curve for the bone marrow compartment (assuming a uniform distribution of blood within the bone marrow). Cumulated activities and residence times for the whole body and bone marrow compartments were then obtained by calculating the area under each respective time activity curve. The target organ in this case is the bone marrow since the goal of dosimetry-guided RAI treatment is to deliver the maximum permissible dose to the patient without permanent damage to the bone marrow. The source organs are the whole primarily (primary in the form of gamma emissions) and the bone marrow (primarily in the form of beta emissions). The absorbed dose to the target organ is the sum of the cumulated activities from the source organs multiplied by their respective S values.

What are some drawbacks of this method? One drawback of the traditional MIRD schema is that the absorbed dose to the tumor is not routinely calculated. Given the wide range of dose variability within a tumor, one cannot be confident (based on bone marrow dosimetry alone) that a lesion will receive a sufficient dose to produce the desired tumoricidal effect. Another limitation revolves around the use of the S values which are derived from anthropomorphic phantoms with uniform activity distribution and defined geometry. Although this is generally acceptable for bone marrow dosimetry (with only two source organs), it is not applicable for lesion-based dosimetry where commonly lesions are encountered with complex shapes and non-uniform distributions of activity. A distinct advantage of I-124 dosimetry lies in its application for lesion-based dosimetry where the higher resolution PET/CT images allow for improved lesion contouring and quantification. Our group demonstrated that there is a significant variation in cumulated activities within individual lesions, whether the lesions depict normal physiologic uptake (as in the salivary glands or thyroid remnants) versus metastatic foci (8). These variations in cumulated activities clearly alter the dosimetric input and cannot be distinguished using traditional I-131 techniques.

For complex lesions which cannot be evaluated using standard anthropomorphic phantoms, voxel-based can be employed, which utilizes or not Monte Carlo based codes. In the simplest voxel-based dosimetry, a lesion (whether it be normal tissue, an individual tumor or a region within a tumor) is divided into voxels of the same 3-dimensional geometry. MIRD formalism is then applied so that the cumulated activity is calculated for each individual voxel comprising the lesion. Each voxel has an assigned S value based on the radioisotope and its location within the lesion, which is assumed to be a homogeneous tissue medium. The absorbed dose to a target lesion is then calculated by summing the products of the cumulated activities and S values of the individual source voxels in a 3-dimensional array (34). The S values are precalculated standarized parameters which do not consider patient-specific tissue heterogeneities. In this case they represent point-dose or voxel-dose kernels. They can be calculated through MC methods (in this case the dosimetry might be considered a type of MC based dosimetry) or through deterministic ones.

MC transport codes can take lesion-based dosimetry to another level by addressing tissue inhomogeneity and assessing their influence on the resulting dose distributions. Target lesions are again divided into iso-volumetric voxels and the cumulated activities are calculated for each individual voxel. However, instead of utilizing S values kernels from standarized uniform tissues, MC based fully developed dosimetries utilize a more complex approach. MC codes can incorporate the probabilistic interactions of radiation with matter including multiple different particles (beta, gamma, photoelectric absorption, Compton scatter and electron-positron pair production) contributing to the absorbed target dose from multiple different pathways that are modeled by computer program computations. MC codes together with appropriate physical data and modeling can provide the most realistic numerical evaluations. Amongst the most evolved, complete and reliable codes of this type are MCNP6 (26), GEANT4 (27,28), and FLUKA (29,30).

## FUTURE I-124 DOSIMETRY CONCEPTS

Areas of future investigation for I-124 dosimetry involve issues related to data collection and data processing. It is widely accepted that 4 time points are required for accurate curve fitting models in order to calculate the cumulated activity. Can this method be streamlined so that fewer time points are needed to generate similar time activity curves with minimal effect on the cumulated activity? This may be plausible given the shorter half life of I-124 as compared to I-131. Another area that our group is focusing on involves the identification of a surrogate for bone marrow activity on anatomical imaging rather than relying on blood sampling. Can a region of interest be drawn on PET/CT (for example around the aorta, vertebral bodies or soft tissues) to simulate the activity within the bone marrow compartment? Our preliminary data indicates that this is likely feasible but a larger patient population is needed for statistical power.

Applying MC codes in I-124 dosimetry is another area of future investigation. MC codes for particle transport pursue the calculation of the random walk, or the track, of (numerical) particles analogous to what would occur in reality. I-124 imaging intrinsically gives information of iodine position and concentration and, therefore, can be used as a surrogate for other iodine isotopes if isotopical correlation and decay correction coefficients are known. Consequently, having iodine distribution defined from I-124 imaging, one can feed the model with decay information of I-124 as well as from other iodine isotopes (considering the mentioned corrections) and obtain dose distributions for isotopes such as I-131.

The combination of MC codes and data from I-124 PET/CT studies can help develop patient- and compound-specific numerical models for 3D dosimetry evaluations (31,32). This type of dosimetry can involve triple voxelized models with resolutions as good as imaging studies allow: voxelized geometry utilizing CT data, voxelized emission distribution utilizing PET data, and voxelized distributions of dose estimators. This means that one can reach optimized resolutions in three aspects: geometry of the regions of interest, source distribution, and detection.

Utilizing this modality, one can assess more realistic particle transport and more representative dose values for large and small regions. The voxelized dose estimators are an important tool for defining hot and cold spots and they provide data for construction of dose-volume histograms or other dosimetry analysis tools. A great advantage of this modality is that it allows for accurate estimation of volume-representative dose values. This means one can determine the volume of influence of a certain dose. For example, if bone marrow is considered, one can determine the maximum, mean, and minimum dose and what fraction of the marrow would receive more than the tolerance dose.

Great contrast appears if this dosimetry modality is compared to previous methods such as those based on the utilization of generic numerical phantoms, e.g. OLINDA/EXM (33), where geometry is not patient specific, organs are considered uniform, emitters are assumed homogeneously distributed in tissues, and doses are whole organ averages. This is also in contrast with more recent modalities based on patient-specific PET/CT images that do not perform the particle transport for each case but utilize standardized fixed point-dose kernels, voxel-dose kernels or other approximations (34). In some cases, the differences are not important but, when heterogeneities are present, large differences might appear. This can be the case for regions with tissue changes or with large activity gradients such as in thyroid remnants.

## CONCLUSIONS

There are a number of ways in which I-124 PET/CT may contribute to current clinical practice in the management of patients with DTC. Studies have shown that I-124 PET/CT is a superior imaging agent as compared to diagnostic I-131 planar whole body scintigraphy with lesion detectability similar to post-treatment I-131 scans. In addition, the potential applications of quantitative I-124 PET/CT imaging for RAI treatment planning have been described that show similar benefits over traditional I-131 dosimetry techniques. However, due to the physical properties of I-124 and its complex decay schema, there remains a need for improved correction methods to ensure the accuracy of diagnostic images and quantitative analysis. Also, there is a need for larger prospective trials to address the number and timing of scans needed for optimal dosimetry protocols. Despite its benefits in lesion detection and measurement of metabolic tumor volumes, for I-124 to be used mainstream, it needs to be more commercially available and at a lower cost.

## Figures and Tables

**Table 1:**
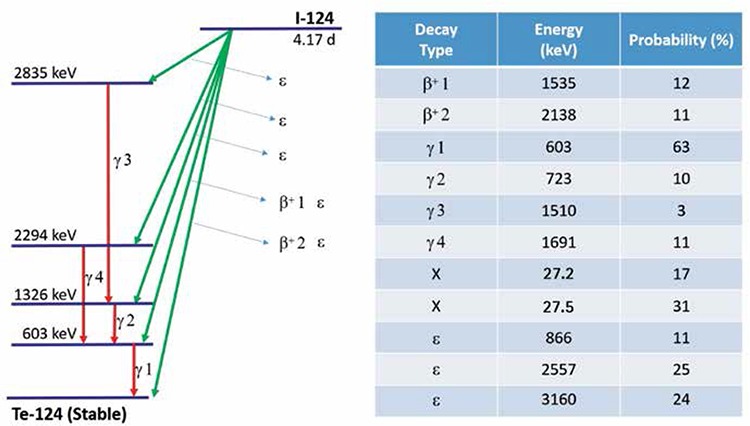
I-124 simplified decay schema and main emissions
